# Soluble Epoxide Hydrolase 2 Expression Is Elevated in Obese Humans and Decreased by Physical Activity

**DOI:** 10.3390/ijms21062056

**Published:** 2020-03-17

**Authors:** Abdelkrim Khadir, Sina Kavalakatt, Dhanya Madhu, Preethi Cherian, Fahd Al-Mulla, Jehad Abubaker, Ali Tiss

**Affiliations:** 1 Biochemistry and Molecular Biology Department, Research Division, Dasman Diabetes Institute, Kuwait City 15462, Kuwait; abdelkrim.khadir@dasmaninstitute.org (A.K.); sina.kavalakatt@dasmaninstitute.org (S.K.); dhanya.madhu@dasmaninstitute.org (D.M.); preethi.cherian@dasmaninstitute.org (P.C.); jehad.abubakr@dasmaninstitute.org (J.A.); 2 Research Division, Dasman Diabetes Institute, Kuwait City 15462, Kuwait; fahd.almulla@dasmaninstitute.org

**Keywords:** EPHX2, sEH, obesity, diabetes, exercise

## Abstract

Epoxide hydrolase 2 (EPHX2) is an emerging therapeutic target in several immunometabolic disorders. EPHX2 metabolizes anti-inflammatory epoxyeicosatrienoic acids into pro-inflammatory diols. The contribution of EPHX2 activity to human obesity remains unexplored. We compared the expression of EPHX2 between lean and obese humans (*n* = 20 each) in subcutaneous adipose tissue (SAT) and peripheral blood mononuclear cells (PBMCs) using RT-PCR, Western Blot analysis, immunohistochemistry, and confocal microscopy before and after a 3-month physical activity regimen. We also assessed EPHX2 levels during preadipocyte differentiation in humans and mice. EPHX2 mRNA and protein expression were significantly elevated in obese subjects, with concomitant elevated endoplasmic reticulum (ER) stress components (the 78-kDa glucose-regulated protein; GRP78, and the Activating transcription factor 6; ATF6) and inflammatory markers (Tumor necrosis factor-α; TNFα, and Interleukin 6; IL6) as compared to controls (*p* < 0.05). EPHX2 mRNA levels strongly correlated with adiposity markers. In obese individuals, physical activity attenuated EPHX2 expression levels in both the SAT and PBMCs, with a parallel decrease in ER stress and inflammation markers. EPHX2 expression was also elevated during differentiation of both human primary and 3T3-L1 mouse preadipocytes. Mediators of cellular stress (palmitate, homocysteine, and macrophage culture medium) also increased EPHX2 expression in 3T3-L1 preadipocytes. Our findings suggest that EPHX2 upregulation is linked to ER stress in adiposity and that physical activity may attenuate metabolic stress by reducing EPHX2 expression.

## 1. Introduction

Soluble epoxide hydrolase 2 (EPHX2), with dual epoxide hydrolase and lipid phosphatase activities, is an emerging therapeutic target in several diseases that share chronic metaflammation as the underlying cause [1]. EPHX2 exerts pro-inflammatory activity by metabolizing anti-inflammatory epoxyeicosatrienoic acids (EETs) into the less active dihydroxyeicosatrienoic acids (DHETs). Together with cyclooxygenase and lipoxygenase, EPHX2 is involved in the arachidonic acid metabolic cascade [2]. One strategy for increasing the action of EETs involves decreasing the activity of EPHX2 [3].

EPHX2 plays further roles in glucose homeostasis, obesity, and diabetes, as shown in rodent and cell-line models [4]. Both the gene deletion and pharmacological inhibition of EPHX2 result in the preservation of islet cells in rodent models of type 1 diabetes and an increase in insulin sensitivity in type 2 diabetes (T2D) models, as does the direct administration of EETs [4]. A study in humans reported that the *EPHX2(K55R)* variant, which results in elevated EPHX2 activity, increases the risk of congenital heart disease in Caucasians [5]. The variant *EPHX2(R287Q)* is associated with an increased risk of obesity and coronary artery calcification in humans [6]. Furthermore, the increased expression and activity of EPHX2 results in decreased EET levels and thus the EPHX2/EET pathway contributes to obesity and diabetes-induced endothelial dysfunction and cardiovascular disease [7].

Plasma EET levels were found to correlate with insulin sensitivity, and carriers of a loss-of-function polymorphism in EPHX2(*R287Q*) demonstrated increased insulin sensitivity, suggesting that EPHX2 inhibition might benefit humans at risk of T2D [8]. In contrast, the same variant was reported to be associated with insulin resistance in Japanese patients with T2D [9]. This discrepancy indicates that factors such as ethnicity, diabetes, obesity, and environment affect the activity of EPHX2 in humans. Nevertheless, the functional contribution of EPHX2 and its anti-inflammatory substrates to human obesity remains poorly understood. Interestingly, gut bacteria were recently reported to be implicated in the regulation of EPHX2 activity [10]. Accordingly, the strong reduction of EPHX2 activity in postprandial states in rats was completely abolished in rats treated with antibiotics to deplete gut bacteria. This effect seems to be insulin-independent and the gut bacteria-derived factors are yet to be elucidated [10].

Physical activity is an important non-pharmacological intervention for managing chronic lifestyle-related diseases, such as obesity and diabetes. While the beneficial effects of exercise are well documented and evidence indicates that regular exercise is a viable, cost effective but underused treatment [11], the related molecular mechanisms are not yet fully understood [12,13]. Reports addressing circulating concentrations of EPHX2 and its metabolites in response to exercise in humans are scarce. Moreover, whether regular exercise affects EPHX2 expression or circulating EET levels in obesity and diabetes is still elusive. A previous report showed that submaximal exercise on a bicycle induced the release of stable DHET metabolites in healthy human volunteers [14]. A more recent study in healthy humans reported that short-term maximal exercise increased the release and accumulation of plasma DHETs but did not alter EPHX2 activity [15]. In addition, exercise-induced vasodilatation in skeletal muscles is reported to release EETs under endothelial nitric oxide synthase inhibition [16].

Given the potential involvement of EPHX2 in obesity and diabetes, we hypothesize that EPHX2 levels in obese humans are elevated over those of normal-weight individuals. This study aims to evaluate the expression levels of EPHX2 in peripheral blood mononuclear cells (PBMCs) and the subcutaneous adipose tissue (SAT) of normal-weight and obese subjects and to investigate the effect of exercise on its expression. We also investigate EPHX2 expression levels during preadipocyte differentiation and the effects of cellular stressors (palmitate and homocysteine) on EPHX2 expression in adipocytes cell lines.

## 2. Results

### 2.1. Characteristics of the Study Population and the Effects of Exercise

The study cohort included 40 subjects without diabetes, of which 20 were of normal weight and 20 were obese. Plasma, PBMCs, and SAT biopsies were collected from subjects. In addition, all members of the obese group were enrolled in a 3-month regular physical activity regimen. Characteristics of the study population at baseline are summarized in Table 1. The obese group displayed significantly higher systolic blood pressure and lower VO_2 max_ compared with the normal-weight (Lean) group (*p* < 0.05). In addition, the former group showed a dysregulated lipid profile, as reflected by higher TG (*p* = 0.008) and lower high-density lipoprotein (HDL) concentrations (*p* = 0.015) as compared to that of the normal-weight group. Obese subjects also showed higher concentrations of glycemic markers (fasting blood glucose, HbA1c, insulin, and HOMA-IR) compared with the normal-weight subjects (*p* < 0.05, Table 1). While the circulating concentrations of inflammatory and stress markers were also elevated in the obese group, only hsCRP and GRP78 concentrations differed significantly (*p* < 0.001 and *p* = 0.012, respectively).

Table 2 summarizes the effect of exercise on the biochemical parameters of subjects in the obese group. After exercise, we observed a significant decrease in adiposity markers (waist circumference and PBF), glycemic index markers (insulin, HOMA-IR, and C-peptide) and systolic blood pressure, along with an increase in VO_2 max_, despite the absence of a prescribed diet. No significant changes were observed after exercise in the metabolic and inflammatory blood markers, except for a significant decrease in insulin and HOMA-IR concentrations (*p* < 0.05).

### 2.2. EPHX2 Expression Levels Are Elevated in the PBMCs and SAT of Obese Subjects and Decrease with Physical Activity

**Figure 1:**
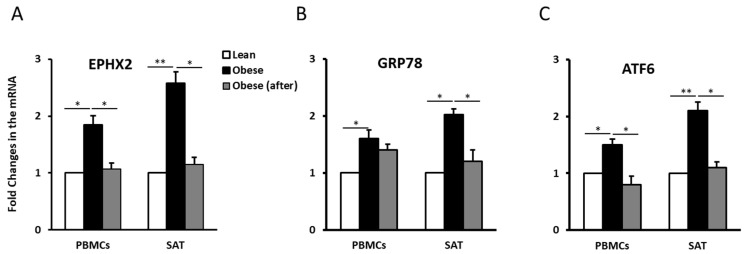
Epoxide hydrolase 2 (EPHX2) mRNA expression in subcutaneous adipose tissue (SAT) and peripheral blood mononuclear cells (PBMCs) from human subjects. mRNA expression of EPHX2 (**A**), the 78-kDa glucose-regulated protein (GRP78) (**B**), and the Activating transcription factor 6(ATF6) (**C**) in PBMCs and SAT of participant groups (lean, obese before exercise, and obese after exercise, *n* = 10 each). mRNA levels were measured by quantitative real-time PCR. Glyceraldehyde 3-phosphate dehydrogenase (GAPDH) was used as an internal control for normalization. Data are presented as fold changes compared with normal-weight participants. * *p* < 0.05; ** *p* < 0.01.

To elucidate the role of EPHX2 in obesity, we assessed its expression levels in PBMCs and SAT in normal-weight and obese individuals before and after exercise. EPHX2 mRNA expression levels were significantly higher in obese than in the normal-weight individuals (*p* < 0.05, Figure 1A). However, EPHX2 expression decreased significantly in the obese group after exercise (*p* < 0.05). Comparable trends were also observed for the ER stress markers GRP78 and ATF6, as their concentrations were elevated in obese subjects and decreased by physical activity (Figure 1B,C). We also observed a trend of higher EPHX2 expression levels in the SAT of obese males than in females; however, these differences did not reach statistical significance (Figure S1).

**Figure 2:**
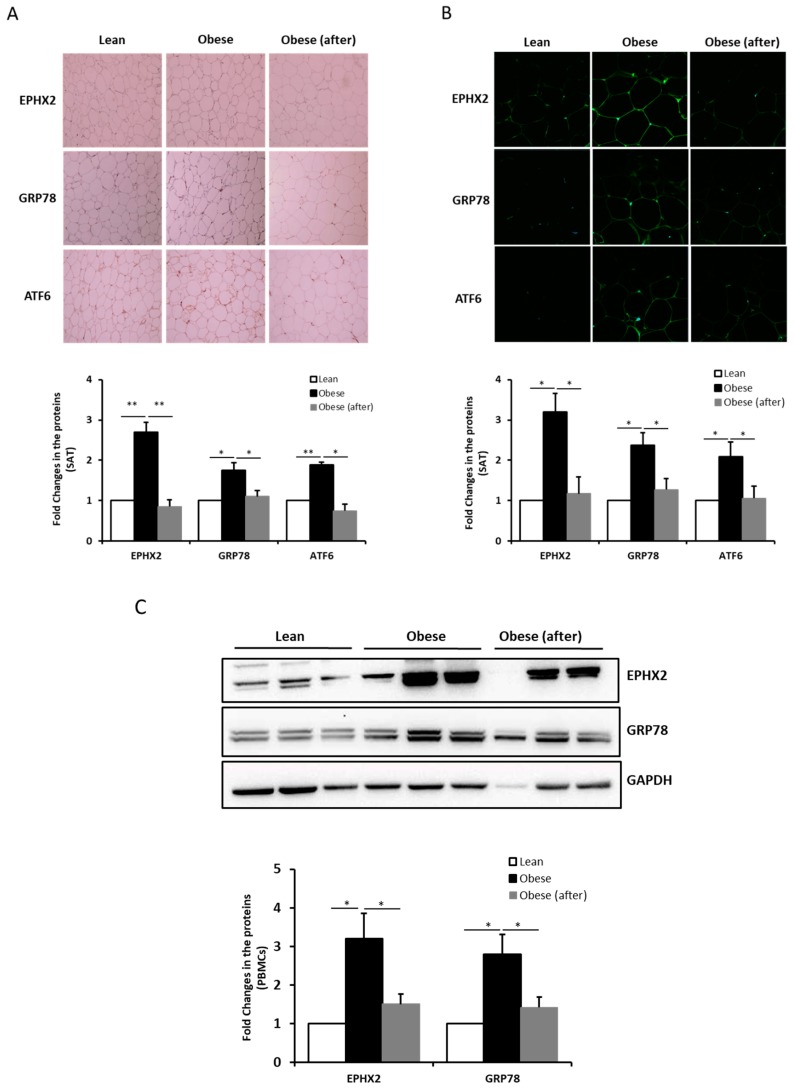
EPHX2 protein levels in SAT and PBMCs from human subjects. Representative immunohistochemical (**A**) and confocal immunofluorescence (**B**) images illustrating EPHX2, GRP78, and ATF6 expression and localization in SAT from lean, obese before exercise, and obese after exercise group (*n* = 5 each). Staining quantification of SAT was performed as detailed in Materials and Methods; data are presented as fold changes compared to the normal-weight group. (**C**) Protein expression of EPHX2, GRP78, and ATF6 in PBMCs according to groups (at least *n* = 9 each). Protein levels were measured by Western Blotting. GAPDH was used as an internal control for normalization. Data are presented as fold changes compared to the lean participants. * *p* < 0.05; ** *p* < 0.01.

We further investigated EPHX2 protein levels in SAT and the effect of exercise on these levels. Both immunohistochemical staining and confocal immunofluorescence revealed more intense EPHX2 staining in obese than in normal-weight SAT (Figure 2A,B). EPHX2 staining was mainly observed in the thin rim of adipocyte cytoplasm. Staining quantification also showed that exercise significantly decreased EPHX2 levels in obese subjects (*p* < 0.05). Comparable trends were also observed for the ER stress markers GRP78 and ATF6, as their expression levels in the SAT were significantly elevated in obesity and decreased after physical activity (*p* < 0.05) (Figure 2A,B). As a control, adiponectin mRNA and protein levels were also assessed in SAT samples from the same subjects; as expected, the converse profile was observed (data not shown).

EPHX2 and GRP78 expression in PBMCs was similar to that of SAT, with elevated levels in obese subjects that decreased after exercise (*p* < 0.05) (Figure 2C); despite some variation between the subjects was observed (Figure S2). ATF6 levels were not quantified due to the unsatisfactory quality of the Western Blot obtained (data not shown).

Spearman correlation analysis of EPHX2 mRNA levels in SAT with demographic and clinical parameters (Table 3) showed that EPHX2 levels correlated positively with adiposity markers (BMI, waist and hip circumferences, and PBF), SBP (*p* < 0.01), and circulating stress markers levels (GRP78 and HSP72) (*p* < 0.05) and negatively with VO_2 max_ (*p* = 0.016).

### 2.3. EPHX2 Expression Levels Increase during Preadipocyte Differentiation

**Figure 3:**
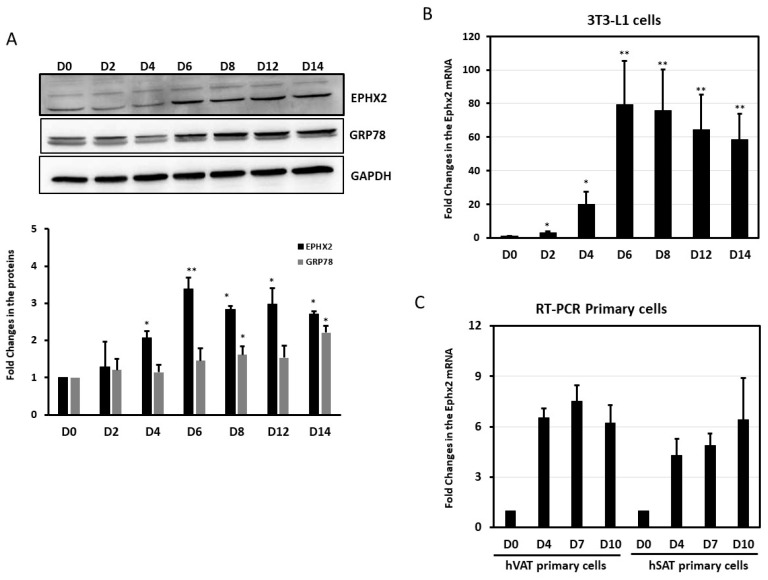
Expression levels of EPHX2 during differentiation of preadipocytes. Protein (**A**) and mRNA (**B**) levels were measured by Western Blot and quantitative real-time PCR, respectively, in 3T3-L1 preadipocytes (D0) and adipocytes differentiated for 14 days (D2–D14) from three independent experiments. (**C**) mRNA levels were measured by quantitative real-time PCR in human visceral (hVAT), subcutaneous (hSAT) preadipocytes (D0), and adipocytes differentiated for 10 days (D4–D10) from two independent experiments. Data are presented as the fold change in differentiated adipocytes compared with preadipocytes (D0). * *p* < 0.05; ** *p* < 0.01.

We assessed the expression levels of EPHX2 during differentiation in murine 3T3-L1 cells and human primary preadipocytes. In 3T3-L1 cells, both EPHX2 mRNA and protein were present in preadipocytes (day 0, D0) and adipocytes (day 14, D14), with expression levels that increased through the differentiation process (Figure 3A,B). Similarly, the levels of EPHX2 mRNA expression in human visceral (hVAT) and subcutaneous (hSAT) adipocytes increased during induced differentiation (day 0–10) (Figure 3C). Preadipocyte differentiation was monitored using O-Red-oil and AdipoRed staining in 3T3-L1 and human primary adipocytes, respectively (Figure S3).

**Figure 4:**
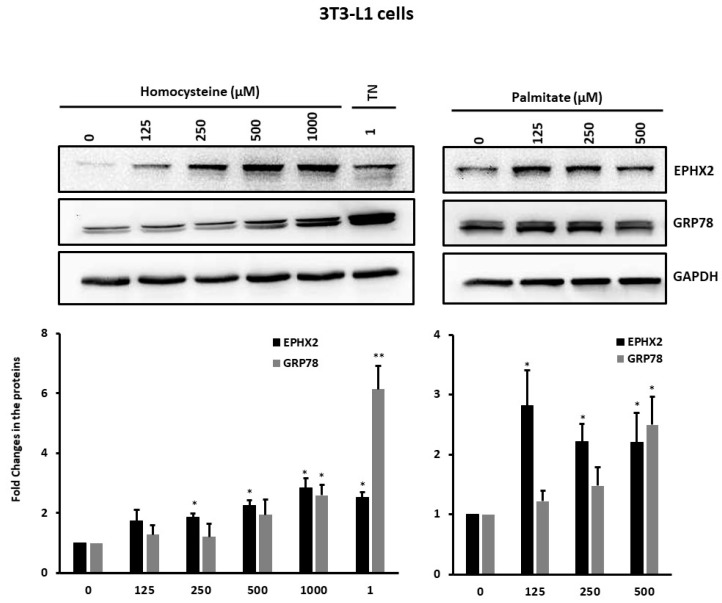
Expression levels of EPHX2 in 3T3-L1 preadipocytes under cellular stressor treatment. The protein levels were measured by Western Blot using 3T3-L1 preadipocytes treated overnight with various concentrations of homocysteine or palmitate or 1 µM Tunicamycin (at least three independent experiments). GRP78 was used as a control for treatment efficacy. * *p* < 0.05; ** *p* < 0.01.

**Figure 5:**
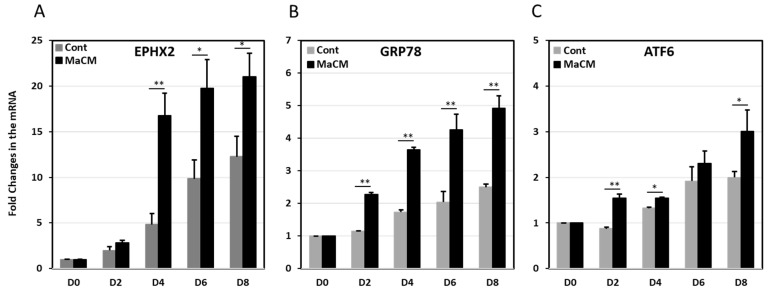
Expression levels of EPHX2 during the differentiation of 3T3-L1 preadipocytes and their treatment with a macrophage culture medium. mRNA expression levels of EPHX2 (**A**), GRP78 (**B**), and ATF6 (**C**) were measured by quantitative real-time PCR in 3T3-L1 preadipocytes (D0) and adipocytes differentiated for 8 days (D8) with and without macrophage-conditioned medium (MaCM). Data are presented as fold changes in differentiated adipocytes compared with preadipocytes from three independent experiments. * *p* < 0.05; ** *p* < 0.01.

We previously reported a different pattern for cellular stress and inflammatory markers in the SAT of normal-weight and obese individuals [17]. This observation, together with the correlation between EPHX2 expression and the cellular stress markers GRP78 and HSP72 observed in the present study, prompted us to investigate the effect of stress and inflammation inducers on EPHX2 expression during preadipocyte differentiation. We observed that the 3T3-L1 cells treated with homocysteine or palmitate displayed concentration-dependent increases in EPHX2 and GRP78 protein expression over those of untreated cells (Figure 4). Finally, 3T3-L1 preadipocytes cultured in macrophage-conditioned media (MaCM) further increased EPHX2 mRNA levels in differentiated adipocytes (*p* < 0.05), GRP78, and ATF6 (Figure 5). Other stress markers, including IRE, PERK and XBP, displayed similar patterns with MaCM treatment (data not shown). The expression levels of genes related to lipogenesis (FASBP4, FASN), lipolysis (LPL, LIPE), inflammation (IL6) as well as C/EBP and PPARγ increased during cell differentiation, confirming that differentiation occurs in both the presence and absence of MaCM.

## 3. Discussion

Inflammation in adipose tissue has been proposed as a key factor in the mechanism underlying obesity-associated metabolic impairments [18]. With their action as immune cells, PBMCs also play a dynamic role in the crosstalk between adipose tissue and other organs in obesity [19]. Here, we observed a correlation between obesity and the expression levels of the pro-inflammatory enzyme EPHX2 in adipose tissue and PBMCs in humans, findings supported by our in vivo results. We observed a significantly elevated expression of EPHX2 and ER stress markers in both the SAT and PBMCs of obese individuals without diabetes as compared to normal-weight individuals. EPHX2 levels strongly correlated with adiposity and stress markers but negatively with a fitness indicator (VO_2 max_). In contrast, a 3-month supervised physical activity regimen significantly attenuated the expression of EPHX2 in both the SAT and PBMCs in obese subjects, with a parallel decrease in ER stress and inflammatory markers. EPHX2 levels also increased during differentiation in both human primary and 3T3-L1 mice preadipocytes. Finally, mediators of cellular stress (palmitate and homocysteine) also induced elevated EPHX2 expression in 3T3-L1 preadipocytes.

EPHX2 activity is considered a major determinant of the bioavailability of anti-inflammatory EET metabolites in the body [20]; thus, targeted inhibition of EPHX2 is an attractive approach to developing therapeutic agents. Numerous studies in animal models and cell lines have reported the involvement of EPHX2 in a variety of metabolic diseases, including cardiovascular diseases (CVD), hypertension, Non-alcoholic fatty liver disease (NAFLD), and diabetes, highlighting the potential benefits of its inhibition [1,21].

While the expression of EPHX2 is normally lower in adipose tissue than in liver or kidney, increased EPHX2 expression and activity were observed in a high-fat-diet-induced mouse model of obesity and during differentiation of murine adipocytes [22]. Consistent with these findings, we observed a marked elevation in EPHX2 levels in SAT of human obese subjects as compared to normal-weight controls. In addition, this increase strongly correlated with adiposity markers (BMI, waist, hip, and PBF), confirming the association between EPHX2 expression and obesity in humans, as observed in mouse models of obesity.

ER stress and inflammatory markers are elevated in the adipose tissue of obese humans [23,24,25]. We report here that the elevated EPHX2 levels in the SAT of obese subjects are accompanied by elevated levels of ER and cellular stress markers (GRP78, ATF6α, and HSP72). Interestingly, the knockdown of GRP78 significantly delayed adipocyte differentiation in 3T3-L1 preadipocytes [26], and GRP78 expression is elevated during adipocyte differentiation in pathological conditions [27] and in the SAT of diabetic patients [23]. The observed elevated levels of HSP72 could be explained by the fact that HSP72 binding increases IRE1α/XBP1 signaling at the ER, thereby inhibiting ER-stress-induced apoptosis [28]. Accordingly, we previously suggested that the increased expression of HSP72 and other HSPs in the SAT and PBMCs of obese non-diabetic subjects is an adaptive response to cope with the ER and cellular stress [29]; this response is attenuated in diabetic subjects. Nevertheless, our results do not address whether the increased EPHX2 expression in our human obese subjects is directly caused by the metaflammation associated with this chronic condition. While inflammation and ER stress may increase EPHX2 expression, EPHX2 inhibition attenuates ER stress in insulin-sensitive tissues, especially the liver [30]. In this context, we showed here that the treatment of 3T3-L1 preadipocytes with classical ER stress (tunicamycin) and metabolic stressors (palmitate and homocysteine) increased EPHX2 expression in a concentration-dependent manner. Furthermore, MaCM treatment during the differentiation of 3T3-L1 cells induced an increased expression of EPHX2 and the ER stress markers GRP78 and ATF6. These data clearly show that metaflammation in adipose tissue plays a key role in the regulation of EPHX2 and ER stress markers and indicate potential contributions by both adipocytes and macrophages to this process.

Our finding of elevated EPHX2 expression in differentiating murine and human preadipocytes is consistent with a previous study reporting decreased EET levels during adipocyte differentiation in vitro and in the adipose tissue of diet-induced obese animals [31]. Our finding further supports the key role of EPHX2 expression and activity in decreasing EET levels; thus, their anti-inflammatory benefits in mature adipocytes as well as in obesity and its related metaflammation. Notably, EPHX2 genetic deficiency or pharmacological inhibition have been shown to attenuate diet-induced ER stress in the liver and adipose tissue of rodents [32,33] and to suppress colonic inflammation induced by obesity [34]. Anti-diabetic drugs such as metformin and GLP-1 analogs, which result in decreased adiposity and cellular stress in humans, might act through decreasing EPHX2 expression or activity, a possibility that remains to be confirmed.

EPHX2 expression is reported to vary between different fat deposits. In epididymal fat isolated from mice, EPHX2 mRNA expression was four times higher in adipocytes than in the stroma [22]. Unfortunately, the volume of the SAT biopsies obtained from our subjects was too low to allow similar comparisons of EPHX2 expression in humans. In addition, we did not have access to visceral adipose deposits from these subjects. However, we suggest that EPHX2 expression levels are likely comparable between human VAT and SAT, as we observed similar EPHX2 levels in human preadipocytes from SAT and VAT and similar elevations in EPHX2 level during the differentiation of those primary cells (Figure 3).

Notably, the expression pattern of EPHX2 was sex-associated, with higher levels of EPHX2 observed in males both in SAT and PBMCs. This observation corroborates the reported sex-related dimorphic regulation of EET bioavailability in circulation and tissues, where both the increased CYP activity (EET synthesis) and decreased EPHX2 expression (EET degradation) observed in females are related to estrogen [35]. Accordingly, the potentiation of CYP/epoxygenase activity compensates for endothelial dysfunction in females, while the estrogen-dependent downregulation of EPHX2 expression yields divergent effects in the circulation (reviewed in [35]).

Physical activity is known to increase anti-inflammatory and stress responses and thus is useful for reducing the risk of chronic metabolic diseases and managing their related complications. Our data show that 3 months of regular physical activity significantly decreased EPHX2 levels in obese subjects, concomitant with decreased PBF, waist circumference, ER stress, and inflammation and increased VO_2 max_. The exercise-induced decrease in EPHX2 expression appeared to be global, as it was observed in both the SAT and PBMCs. Assessing EPHX2 expression in other tissues would be of interest. Interestingly, previous studies have reported that EET treatment prevented obesity-induced cardiomyopathy in db/db mice by increasing VO_2 max_, with a concomitant decrease in VCO_2_/VO_2_, and exerted other metabolic effects [36]. In line with this improved oxygen consumption, mice fed a high-fat/high-fructose diet showed higher VO_2 max_ and greater weight loss after treatment with the EPHX2 inhibitor AR9281 [37].

The mechanism underlying the effect of exercise on EPHX2 expression is yet to be elucidated. The few previous studies of this relationship focused on the effect of exercise on EET availability but not on EPHX2. One such study used the EPHX2 inhibitor 1-trifluoromethoxyphenyl-3-(1-propionylpiperidin-4-yl) urea (TPPU) in mice, reporting that an exercise-induced increase in EET expression exerts cardioprotection [38]. Another study reports lower EET levels in activity-restricted adult male rhesus macaques as compared to those with normal activity, which is likely due to higher EPHX2 activity [39]. In response to exercise, human plasma DHET levels are reported to increase in healthy subjects [14,15]. However, these studies provided no evidence that this effect resulted from impaired EPHX2 expression or activity. The only study focusing on EPHX2 modulation by exercise was reported in mice and showed decreased EPHX2 expression only in the kidney [40]. We and others have previously reported the beneficial effects of regular exercise in obese individuals [17,41,42]. Here, our exercising obese group showed increased maximal oxygen consumption along with improvements in the metabolic markers HDL, TG, glycemia, and stress response chaperones (GRP78 and HSP72), suggesting that the effects of our exercise protocol were global despite the negligible weight decrease and absence of a specific prescribed diet.

The present study is the first to report the effect of exercise on EXPH2 expression in obese humans, including the determination of the pattern of EPHX2 expression during adipocyte differentiation and under metabolic stress. Despite our novel findings, this study has some limitations. First, we did not have access to visceral adipose tissue biopsies, which would be more relevant to the pathophysiology of obesity and diabetes. Second, despite improvements in several fitness and metabolic markers, we cannot exclude the possibility that the lack of controlled dietary intake may have affected our interpretation of the EPHX2 levels and the efficacy of physical activity. Furthermore, the limited number of study participants does not allow for the generalization of our findings. Finally, we did not consider the potential contribution of the EPHX2 gene polymorphism in our data analysis, as we did not have access to this information. However, the strengths of this study include the involvement of a high-risk group of obese adults without T2D who underwent a supervised moderate exercise protocol as a behavioral approach to improve global health without diet restriction. Further studies are required to understand the crosstalk between EPHX2 levels and insulin resistance and the role of EPHX2 in adipose tissue, including its status in VAT. These studies will help to elucidate the mechanisms by which EPHX2 affects adiposity, insulin resistance, and diabetes.

## 4. Materials and Methods

### 4.1. Study Population

A total of 20 normal-weight (lean, 20 ≤ BMI < 25 kg/m^2^) and 20 obese (30 ≤ BMI < 40 kg/m^2^) adult individuals without diabetes were recruited to the study through the clinics of the Dasman Diabetes Institute (DDI), Kuwait. The protocol was approved by the Institutional Review Board of the DDI and was conducted in line with the principles of the Declaration of Helsinki. Written informed consent was obtained from all participants before enrollment in the study. Subjects with a history of major illness or medications or who performed regular physical activity within the last 6 months before study commencement were excluded.

### 4.2. Exercise Protocol and Anthropometric Measurements

Obese subjects were enrolled in a supervised exercise program at the Medical Fitness Center (MFC) of the DDI, as previously reported [43]. Before the first exercise session, each subject underwent the “CPET” cardiopulmonary exercise test (COSMED, Quark, Italy), which uses an ergometer to measure the maximum heart rate (max HR) and maximum oxygen consumption (VO_2 max_) during use of an electromagnetically-braked cycle. The exercise program combined resistance training with treadmill or cycling and moderate-intensity aerobic exercise. Each exercise session included 10 min of warming up and 10 min of cooling down at 50%–60% max HR in addition to the 40 min of described exercise at 65–80% max HR. Subjects exercised 3 times/week for a period of 3 months under the supervision and monitoring of fitness professionals at the MFC to maintain the recommended HR during the training. The effectiveness of the exercise regimen was assessed at the end of the 3-month program by comparison with parameters at the baseline.

### 4.3. Blood and Tissue Sampling

Venous peripheral blood and SAT biopsies were obtained at baseline and after the 3-months of exercise and were processed as previously reported [44]. Briefly, plasma and serum were prepared using vacutainer tubes for clinical analysis, and aliquots were stored at −80 °C. PBMCs were prepared from fresh blood samples collected in Ethylenediamine tetraacetic acid (EDTA) tubes. PBMCs were isolated using Ficoll–Hypaque density-gradient centrifugation. SAT biopsies specimens (~300 mg) were obtained from the periumbilical area by surgical biopsy after local anesthesia. The specimens were then rinsed in cold PBS, divided into 4 pieces, and stored appropriately until assayed.

### 4.4. Blood Biochemistry

Concentrations of blood clinical markers and plasma inflammatory and metabolic markers were measured as previously reported [44]. Briefly, the HbA1c level was determined using the variant device (Bio-Rad, Hercules, CA, USA). Glucose and lipid concentrations were measured on the Siemens Dimension RXL chemistry analyzer (Diamond Diagnostics, Holliston, MA, USA). Plasma concentrations of inflammatory markers were measured using a Bioplex-200 multiplexing technology system (BioRad, Hercules, CA, USA). High-sensitivity CRP (hsCRP) concentrations were determined by ELISA (Biovendor ELISA kit, Asheville, NC, USA). Circulating GRP78 and HSP72 protein concentrations were determined in plasma using the human ELISA kit (Enzo LifeSciences, Lausen, Switzerland). All assays were performed according to the manufacturers’ instructions.

### 4.5. Immunohistochemical and Immunofluorescence Analyses

Formalin-fixed, paraffin-embedded SAT sections were used for immunohistochemical analysis and immunofluorescence investigations (*n* = 5 each), as previously described [23]. For the immunohistochemical analysis, we used anti-GRP78 (Abcam, Cambridge, MA, USA), Imgenex anti-ATF6 (Novus, Littleton, CO, USA), and anti-EPHX2 antibodies (OriGene Technologies, Inc., Rockville, MD, USA). The immunohistochemical analysis results were quantified using ImageScope software version 11.1 (Aperio, Vista, CA, USA), as previously reported [29]. For immunofluorescent staining, tissue sections were incubated with anti-GRP78 or anti-ATF6 antibody conjugated with Alexa Fluor-488 (Bioss, Woburn, MA, USA). EPHX2 tissue sections were incubated with anti-EPHX2 antibody (OriGene Technologies, Inc., Rockville, USA) and then incubated with Alexa Fluor-488–conjugated goat anti-mouse secondary antibody (Molecular Probes, ThermoFisher, USA). Nuclei were stained using 0.05% DAPI. The sections were viewed with a Zeiss LSM 710 confocal laser-scanning microscope, and representative areas of the sections were photographed under a 40× objective.

### 4.6. Cell Cultures

Mouse preadipocytes (3T3-L1) and macrophages (RAW264.7) were obtained from American Type Culture Collection (ATCC) (Manassas, VA, USA), cultured, and induced to differentiate in a growth medium as previously reported [17]. For indirect co-culture of 3T3L1 and RAW264.7 cells, media from RAW264.7 cells were collected and sterile filtered (0.2 μm). The differentiation of 3T3-L1 cells was induced using MaCM supplemented with a differentiation cocktail (1% PS, 10% bovine calf serum (BCS), 0.5 mM 3-isobutyl1-methylxanthine, 1μM dexamethasone, and 1μg/mL insulin) for 2 days. The medium was then changed to MaCM with 10% BCS and insulin (1μg/mL) and was replaced every 2 days through day 8. At least 3 independent experiments were performed for each condition. Human primary preadipocyte cells from hSAT and hVAT (#PT5001 and #PT5005, Lonza, Walkersville, MD, USA) were processed and differentiated according to the supplier’s instructions in 2 independent experiments.

### 4.7. Quantitative Real-Time PCR

Total RNA was extracted from SAT (*n* = 10 each subject group) using the RNeasy Lipid Tissue MiniKit (Qiagen, Hilden, Germany). Total RNA from PBMCs (*n* = 10 each subject group), cell lines (3 independent experiments), and primary adipocytes (2 independent experiments) was extracted using TRIZOL reagent. cDNA was synthesized from total RNA samples using the High Capacity cDNA Reverse Transcription Kit (Applied Biosystems, Foster City, CA, USA). Conventional qRT-PCR was performed using the Applied Biosystem7500 system, and gene expression was normalized to that of GADPH. The primers used are presented in Table S1. Differences in gene expression between groups were assessed using the ΔΔCT method.

### 4.8. Western Blot Analysis

Western Blot analysis was performed using PBMCs from lean and obese subjects (at least *n* = 9 for each group), differentiated 3T3-L1 cells, and 3T3-L1 preadipocytes as previously reported [43] (3 independent experiments). Briefly, whole cell extracts were prepared in RIPA buffer, and protein concentration was determined using the Bradford method using β-globulin as a standard. Protein samples (20 µg) were loaded and resolved on 12% SDS-PAGE gels. After transfer to PVDF membranes, the proteins were probed with anti-GRP78 (Abcam, Cambridge, MA, USA) and anti-EPHX2 antibodies (OriGene Technologies, Inc., Rockville, MD, USA) overnight at 4 °C. After washing, the membranes were incubated with rabbit horseradish-peroxidase-conjugated secondary antibody for 2 h at room temperature and the proteins visualized using super sensitivity West Femto-ECL reagent (Thermo Scientific, Waltham, MA, USA). Protein bands were visualized by chemiluminescence and the images captured using the Versadoc 5000 system (Bio-Rad), and the bands intensity was determined using Quantity One Software (Bio-Rad). GAPDH was used as an internal control for protein loading and was detected with an anti-GAPDH antibody (ab2302; Millipore, Temecula, CA, USA).

### 4.9. Oil Red O Staining

3T3-L1 cells were washed twice with PBS, fixed with 10% formalin for 1 h at RT, washed twice again with PBS, and incubated with 60% isopropanol for 5 min. Finally, cells were incubated with 0.3% Oil Red O solution for 20 min. Coverslips were washed with distilled water five times and mounted onto a slide. Images were captured using a panoramic digital slide scanner.

### 4.10. AdipoRed Assay

Human primary preadipocyte cells from hSAT and hVAT were plated and left overnight to attach. Then, AdipoRed Assay Reagent (Lonza, Walkersville, MD, USA) was used to stain the cells following the manufacturer’s instructions. Images were captured using a digital camera.

### 4.11. Statistical Analysis

Statistical analyses were performed using SPSS software (v25.0; SPSS Inc., IL, USA). Descriptive statistics are reported here as the mean ± standard error. The Chi-square test was used for categorical variables, and the Wilcoxon non-parametric *t*-test was used for skewed and continuous variables. The paired *t*-test was used to determine the significance of differences between means within the obese group before and after exercise. Spearman’s correlation coefficient was used to assess the correlations between variables. *p* < 0.05 was considered statistically significant.

### 4.12. Ethics Approval and Consent to Participate

Written informed consent was obtained from all participants prior to starting this study (RA-2010-003, 18 May 2010), which was approved by the Review Board of Dasman Diabetes Institute and was conducted in line with the principles of the Declaration of Helsinki.

## Figures and Tables

**Table 1:** Physical, clinical, and biochemical characteristics of the cohort study at baseline.

Marker	Lean (*n* = 20) (Mean ± SD, *n* = 20)	Obese (*n* = 20) (Mean ± SD, *n* = 20)	(*p*-Value)
**Anthropometric and physical characteristics**			
AGE (year)	38.75 ± 9.60	40.13 ± 8.68	0.55
Gender (M/F)	8/12	10/10	0.37
BMI (kg/m^2^)	22.72 ± 2.09	34.83 ± 2.99	<0.001
Waist (cm)	79.45 ± 15.96	108.52 ± 13.71	<0.001
Hip (cm)	92.07 ± 14.98	118.15 ± 8.29	<0.001
PBF (%)	27.50 ± 5.35	39.36 ± 5.12	<0.001
SBP (mmHg)	113.00 ± 10.81	127.50 ± 11.89	0.01
DBP (mmHg)	76.43 ± 6.33	82.00 ± 10.14	0.13
V_O2, Max_ (mL/kg/min)	21.63 ± 3.76	16.54 ± 4.83	0.03
**Metabolic markers**			
Cholesterol (mmol/L)	5.22 ± 0.91	5.38 ± 1.10	0.74
HDL (mmol/L)	1.48 ± 0.53	1.19 ± 0.26	0.015
LDL (mmol/L)	3.23 ± 0.91	3.39 ± 0.98	0.28
TG (mmol/L)	0.91 ± 0.42	1.47 ± 0.83	0.008
Glucose (mmol/L)	5.0 ± 0.5	5.7 ± 0.7	0.013
HbA1c (%)	5.50 ± 0.44	5.93 ± 0.48	0.014
Insulin (ng/mL)	2.6 ± 1.3	4.2 ± 2.5	0.029
HOMA-IR	0.7 ± 0.5	1.1 ± 0.7	0.025
**Inflammatory markers**			
IL-6 (pg/mL)	17.06 ± 2.9	17.40 ± 4.4	0.39
TNF-a (pg/mL)	123.52 ± 35	126.47 ± 43	0.94
hsCRP (μg/mL)	1.7 ± 1.2	5.3 ± 3.6	<0.001
**Stress markers**			
GRP78 (μg/mL)	0.925 ± 0.95	1.2 ± 0.75	0.012
HSP72 (ng/mL)	1.85± 0.53	1.62 ± 0.45	0.055

Abbreviations: ATF6, activating transcription factor 6; BMI, body mass index; DBP, diastolic blood pressure; EPHX2, epoxide hydrolase 2; GRP78, 78-kDa glucose-regulated protein; HbA1c, hemoglobin A1c; HDL, high-density lipoprotein; HOMA-IR, homeostatic model assessment of insulin resistance; hsCRP, high-sensitivity CRP; HSP72, heat shock protein 72; IL-6, interleukin 6; LDL, low-density lipoprotein; PBF, percent body fat; SBP, systolic blood pressure; TGL, triglyceride; TNF-α, Tumor necrosis factor-α; VO_2,max_, maximum oxygen consumption.

**Table 2:** Physical, clinical, and biochemical characteristics of obese subjects without diabetes before and after exercise.

Marker	Obese before (Mean ± SD, *n* = 20)	Obese after (Mean ± SD, *n* = 20)	*p*-Value
**Anthropometric and physical characteristics**			
BMI (kg/m^2^)	34.83 ± 2.99	34.08 ± 3.31	0.09
Waist (cm)	108.52 ± 13.71	105.17 ± 10.97	0.03
Hip (cm)	118.15 ± 8.29	116.57 ± 9.05	0.23
PBF (%)	39.36 ± 5.12	38.44 ± 5.09	0.02
SBP (mmHg)	127.50 ± 11.89	117.22 ± 8.20	0.01
DBP (mmHg)	82.00 ± 10.14	79.39 ± 4.95	0.08
VO_2,max_ (mL/kg/min)	16.54 ± 4.83	18.94 ± 3.78	0.01
**Metabolic markers**			
Cholesterol (mmol/L)	5.38 ± 1.10	5.37 ± 1.22	0.83
HDL (mmol/L)	1.19 ± 0.26	1.29 ± 0.49	0.11
LDL (mmol/L)	3.39 ± 0.98	3.47 ± 1.13	0.69
TG (mmol/L)	1.47 ± 0.83	1.28 ± 0.77	0.17
Glucose (mmol/L)	5.7 ± 0.7	5.5 ± 0.6	0.49
HbA1c (%)	5.93 ± 0.48	5.78 ± 0.42	0.28
Insulin (ng/mL)	4.2 ± 2.5	3.30 ± 1.11	0.01
HOMA-IR	1.1 ± 0.7	0.8 ± 0.2	0.04
**Inflammatory markers**			
IL-6 (pg/mL)	17.4 ± 4.4	16.6 ± 6.5	0.87
TNF-a (pg/mL)	126.4 ± 43	123.8± 33.1	0.59
hsCRP (μg/mL)	5.3 ± 3.6	6.2 ± 4.1	0.43
**Stress markers**			
GRP78 (μg/mL)	1.2 ± 0.75	0.85 ± 0.26	0.02
HSP72 (ng/mL)	1.62± 0.45	2.08 ± 0.59	0.35

Abbreviations: ATF6, activating transcription factor 6; BMI, body mass index; DBP, diastolic blood pressure; EPHX2, epoxide hydrolase 2; GRP78, 78-kDa glucose-regulated protein; HbA1c, hemoglobin A1c; HDL, high-density lipoprotein; HOMA-IR, homeostatic model assessment of insulin resistance; hsCRP, high-sensitivity CRP; HSP72, heat shock protein 72; IL-6, interleukin 6; LDL, low-density lipoprotein; PBF, percent body fat; SBP, systolic blood pressure; TGL, triglyceride; TNF-α, Tumor necrosis factor-α; VO_2,max_, maximum oxygen consumption.

**Table 3:** Spearman correlation with EPHX2 mRNA.

Markers	R-Value	*p*-Value
BMI (kg/m^2^)	0.73	0.001
Waist (cm)	0.66	0.008
Hip (cm)	0.51	0.004
PBF (%)	0.54	0.002
SBP (mmHg)	0.49	0.006
DBP (mmHg)	0.30	0.110
VO_2, Max_ (mL/kg/min)	–0.44	0.016
HSP72	0.43	0.020
GRP78	0.52	0.002

Abbreviations: BMI, body mass index; DBP, diastolic blood pressure; EPHX2, epoxide hydrolase 2; GRP78, 78-kDa glucose-regulated protein; HSP72, heat shock protein 72; PBF, percent body fat; SBP, systolic blood pressure; VO_2,max_, maximum oxygen consumption.

## Supplementary Materials

The following are available online at https://www.mdpi.com/1422-0067/21/6/2056/s1.

## Abbreviations

DBP	diastolic blood pressure
EPHX2	epoxide hydrolase 2
HbA1c	hemoglobin A1c;
HDL	high-density lipoprotein
HR	heart rate
hsCRP	high-sensitivity CRP
LDL	low-density lipoprotein;
PBF	percent body fat
SAT	subcutaneous adipose tissue
PBMC	peripheral blood mononuclear cell;
sEH	soluble epoxide hydrolase;
SBP	systolic blood pressure;
TGL	triglyceride
VO_2,max_	maximum oxygen consumption;
WC	waist circumference.
