# Supplementary material for: Soluble Epoxide Hydrolase 2 Expression Is Elevated in Obese Humans and Decreased by Physical Activity
Source: Int J Mol Sci. 2020 Mar 17;21(6):2056. doi: 10.3390/ijms21062056 (PMC7139757; doi:10.3390/ijms21062056)
Supplement: Supplementary file 1 [file ijms-21-02056-s001.zip › ijms-718379-supplementary.pptx]

## Slide 1
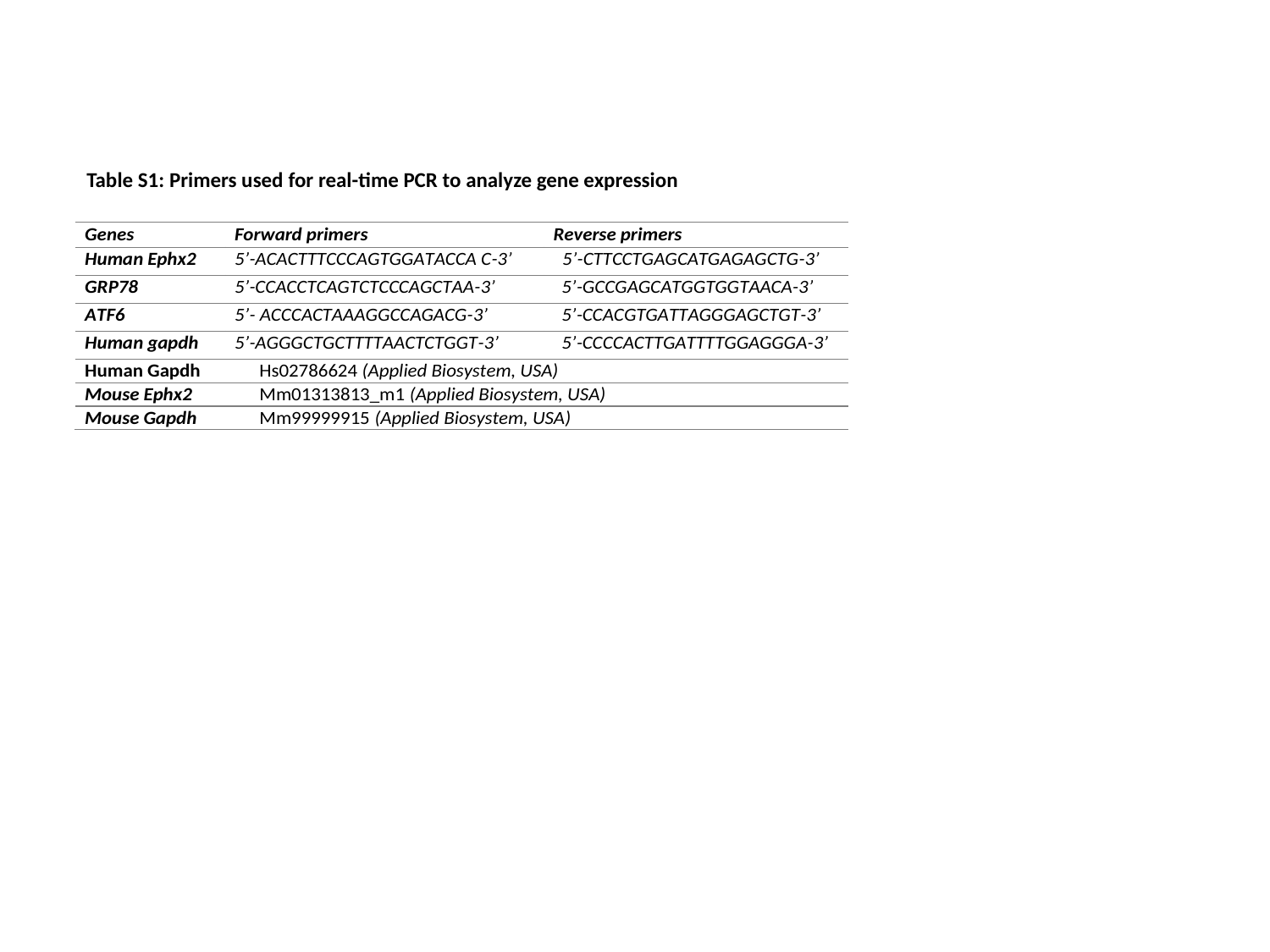

Table S1: Primers used for real-time PCR to analyze gene expression

## Slide 2
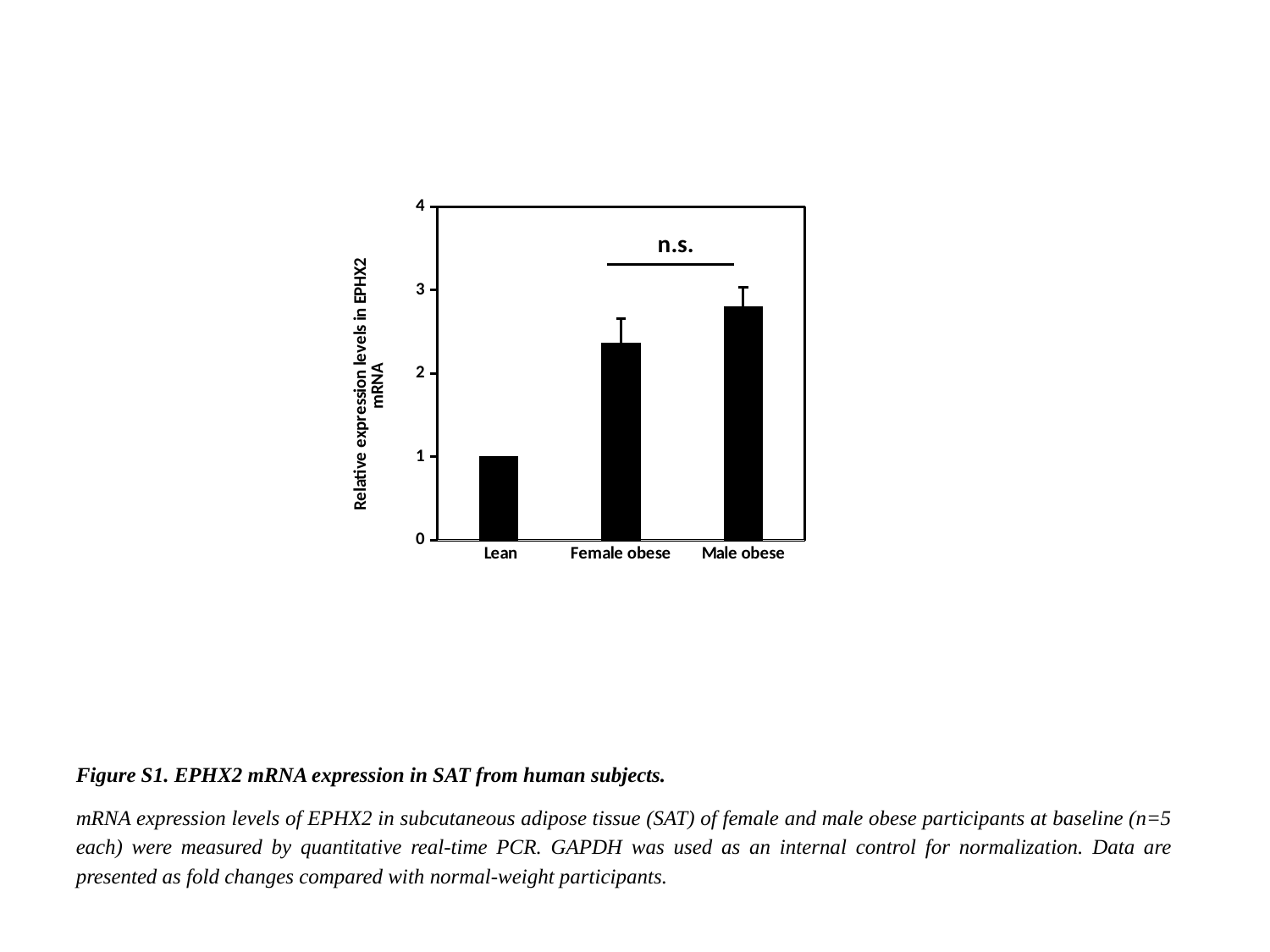

### Chart
| Category | RT-PCR AT |
|---|---|
| Lean | 1.0 |
| Female obese | 2.3604651162790695 |
| Male obese | 2.802325581395349 |n.s.
Figure S1. EPHX2 mRNA expression in SAT from human subjects.
mRNA expression levels of EPHX2 in subcutaneous adipose tissue (SAT) of female and male obese participants at baseline (n=5 each) were measured by quantitative real-time PCR. GAPDH was used as an internal control for normalization. Data are presented as fold changes compared with normal-weight participants.

## Slide 3
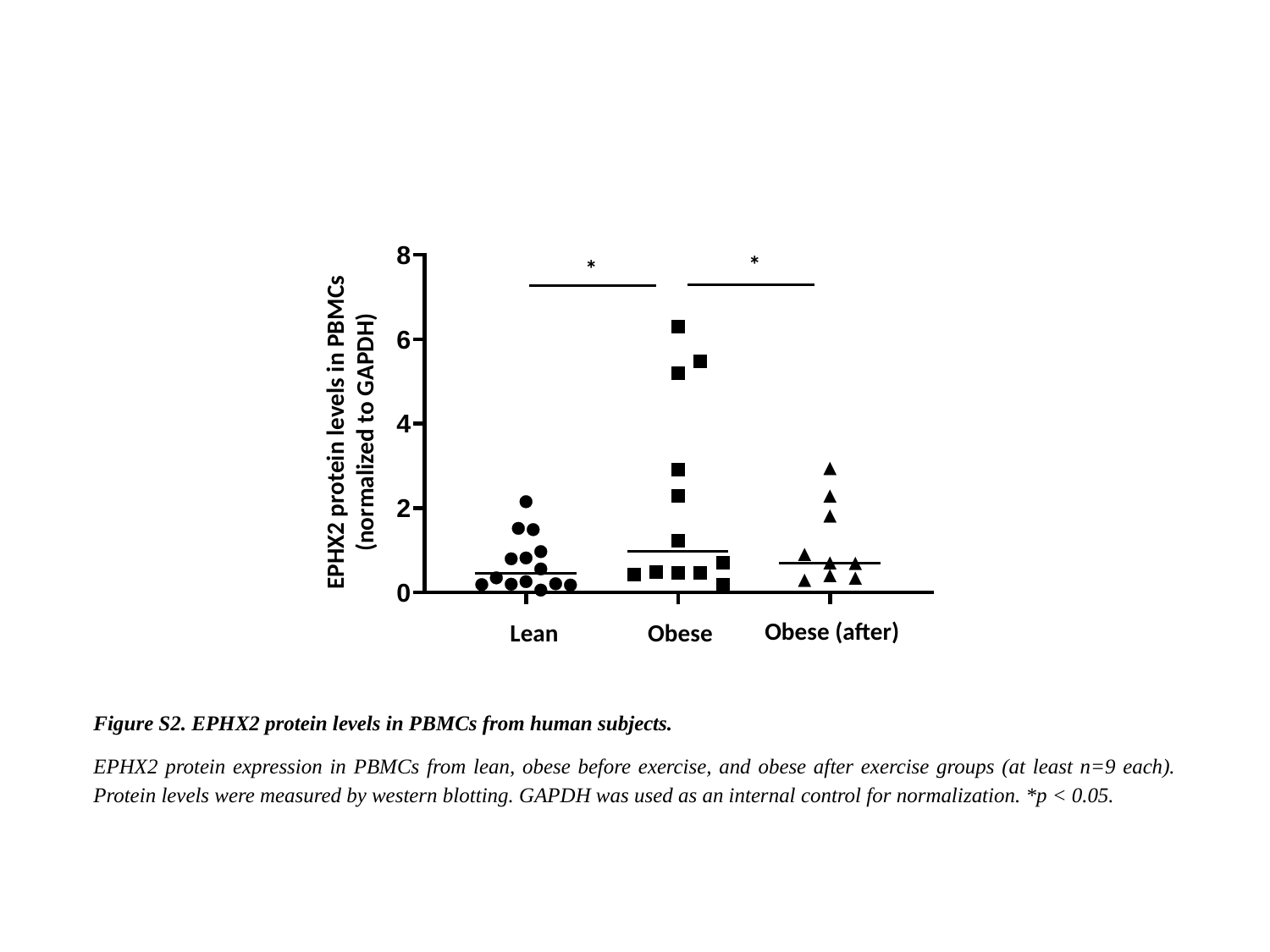

*
*
EPHX2 protein levels in PBMCs
(normalized to GAPDH)
Obese (after)
Lean
Obese
Figure S2. EPHX2 protein levels in PBMCs from human subjects.
EPHX2 protein expression in PBMCs from lean, obese before exercise, and obese after exercise groups (at least n=9 each). Protein levels were measured by western blotting. GAPDH was used as an internal control for normalization. *p < 0.05.

## Slide 4
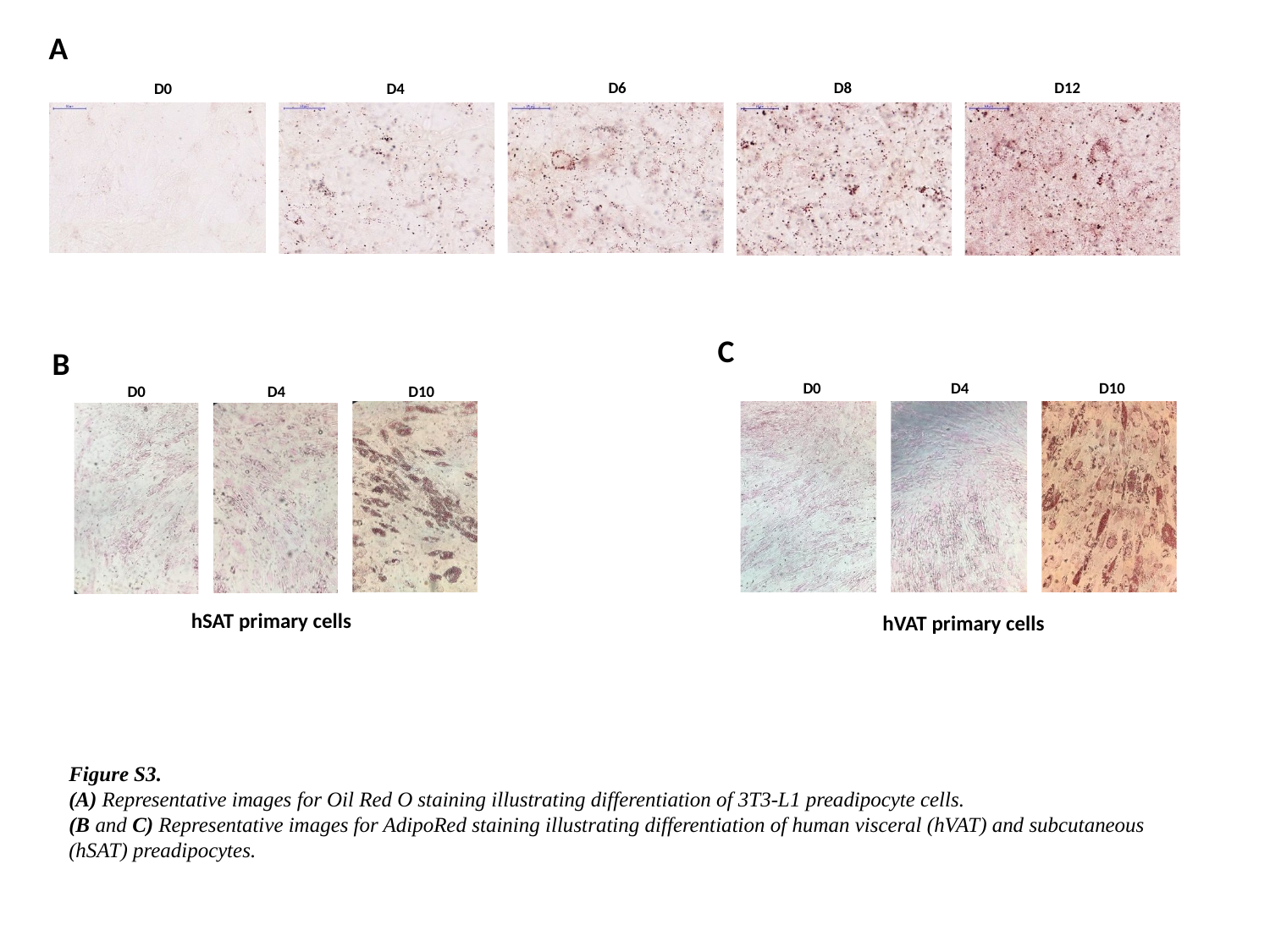

A
D8
D12
D6
D0
D4
C
B
D0
D4
D10
hVAT primary cells
D0
D4
D10
hSAT primary cells
Figure S3.
(A) Representative images for Oil Red O staining illustrating differentiation of 3T3-L1 preadipocyte cells.
(B and C) Representative images for AdipoRed staining illustrating differentiation of human visceral (hVAT) and subcutaneous (hSAT) preadipocytes.
